# Intracystic gastrointestinal stromal tumor developed in the round ligament of the liver

**DOI:** 10.1016/j.radcr.2024.04.067

**Published:** 2024-05-14

**Authors:** Naoki Kataoka, Shoji Oura, Akito Furuta

**Affiliations:** a Department of Surgery, Kishiwada Tokushukai Hospital, Kishiwada-city, Japan; b Department of Gastroenterology, Kishiwada Tokushukai Hospital, Kishiwada-city, Japan

**Keywords:** Gastrointestinal stromal tumor, GIST, Intracystic GIST, Round ligament of the liver

## Abstract

A 44-year-old woman was referred to our hospital for the examination and treatment of a presumed gallbladder tumor. Both ultrasound and computed tomography showed an intracystic tumor but failed to point out the discontinuity between the cystic lesion and the gallbladder. Magnetic resonance imaging, however, could clearly depict the presumed intracystic tumor and the discontinuity between the gallbladder and the target lesion. Both contents of the gallbladder and the cystic lesion showed hypo and hyper intense patterns, though both with slightly different intensities, on T1- and T2-weighted images, respectively. Under the preoperative diagnosis of early gallbladder cancer despite these image findings, laparoscopic cholecystectomy was attempted to the patient. Laparoscopic observation, however, revealed that the target lesion was not continuous with the gallbladder and was located in the round ligament of the liver. Intraoperative findings led us to do cholecystectomy and resection of the adjacent cystic tumor. The intracystic tumor was 3 cm in size and had minute solid component inside the cyst wall. Pathological study of the presumed gallbladder cancer showed epithelioid cells and spindle cells growing in sheet like and storiform fashions, respectively. Cystic walls mainly consisted of hypo cellular fibrous components. Immunohistochemical staining of the tumor was positive for CD117 and negative both for desmin and S100, leading to the diagnosis of gastrointestinal stromal tumor. MIB-1 labelling index of the gastrointestinal stromal tumor was 8%. The patient recovered uneventfully and has been well without any recurrences for 3 months.

## Introduction

Gastrointestinal stromal tumors (GISTs) are rare solid malignancies in the gastrointestinal (GI) tract but are the most common mesenchymal tumor in GI tract [1], [2], [3]. GISTs likely originate from the interstitial cells of Cajal (ICCs) and carry a mutation in the Kit gene in approximately 80% of cases [4,5]. ICCs are regarded as the pacemakers of the GI tract [6], being consistent with the fact that GISTs often occur in organs with high motility in the gastrointestinal tract, e.g., stomach and duodenum. Rare cases, however, have been reported to occur in the extra-GI tract such as mesentery and omentum [7].

The round ligament of the liver is a cord tissue that worked as an umbilical vein during the fetal period, but loses its function after birth and only acts as a supporting tissue to the liver. The round ligament, therefore, hardly develop any disorders except for fibrous tumors and mesotheliomas.

Gallbladder tumors are the most common neoplastic disease that occurs in the hepatic hilum. Diagnostic physicians generally tend to focus on whether the neoplastic lesion is benign or malignant. However, when a cystic lesion in contact with the gallbladder occurs at the hepatic hilum, it is often difficult to negate even the continuity between the gallbladder and the cystic lesion due to anatomical characteristics.

We herein report an intracystic GIST developed in the round ligament of the liver that was improperly diagnosed as a gallbladder mass preoperatively.

## Case report

**Fig. 1:**
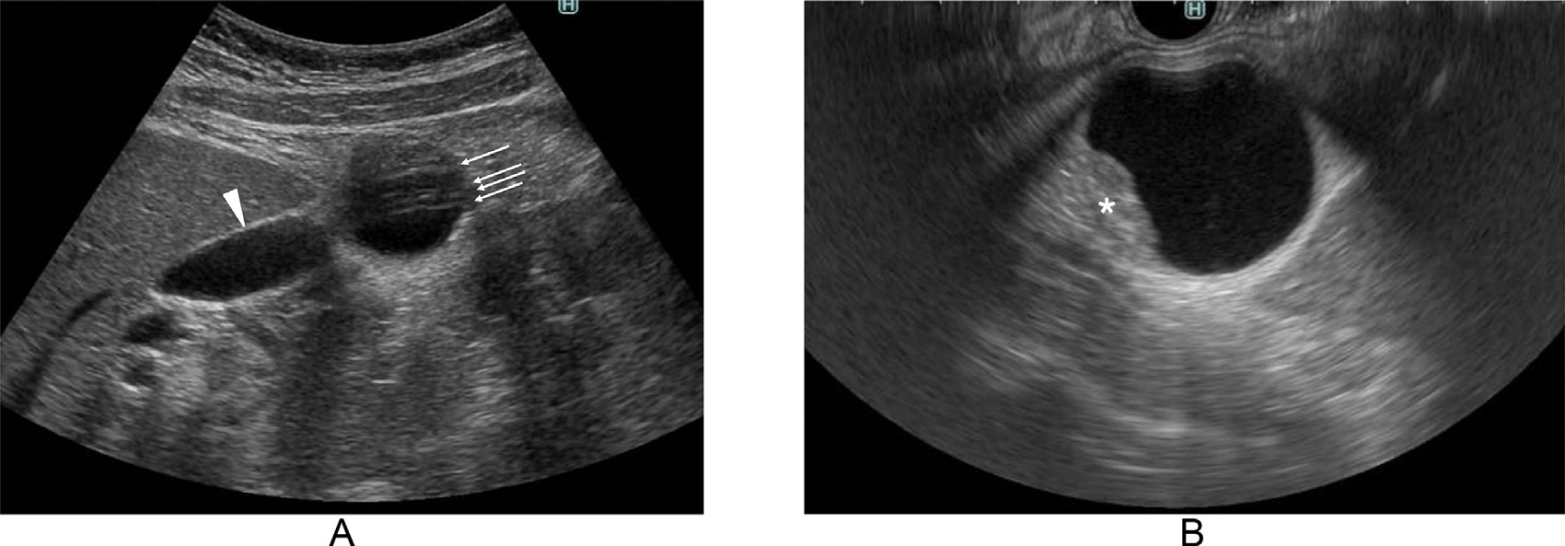
Ultrasound findings. (A) Ultrasound could not negate the continuity between the gallbladder (arrowhead) and the cystic lesion containing the obscured solid part. The presence of multiple reflections of ultrasound (arrows) further made it difficult to evaluate the continuity between the 2. (B) Endoscopic ultrasound clearly showed the intra-cystic solid tumor (asterisk).

**Fig. 2:**
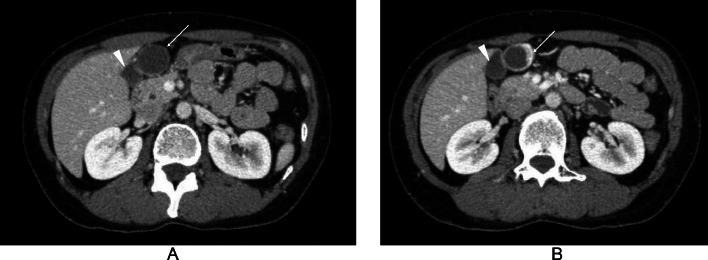
Computed tomography (CT) findings. (A) CT findings could not negate the continuity between the gall bladder (arrowhead) and the cystic lesion (arrow). (B) CT clearly showed an early enhancement pattern in the solid part (arrow) of the intracystic tumor.

**Fig. 3:**
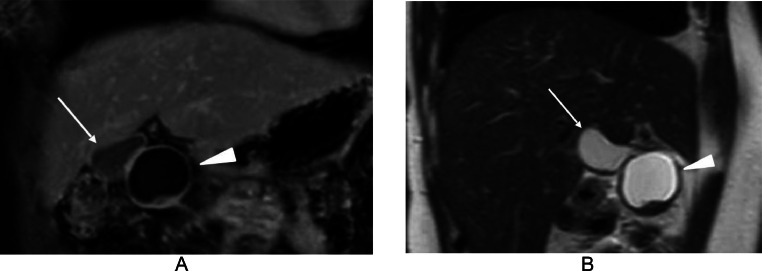
Magnetic resonance imaging (MRI) findings. (A) Coronal view MRI of both the gallbladder (arrow) and intracystic tumor (arrowhead) contents showed a hypo-intense pattern on T1-weighted images. The latter intensity was much lower than that of the former. (B) Sagittal view MRI of the 2 contents showed a hyper-intense pattern on T2-weighted images. The latter intensity was somewhat higher than that of the former.

**Fig. 4:**
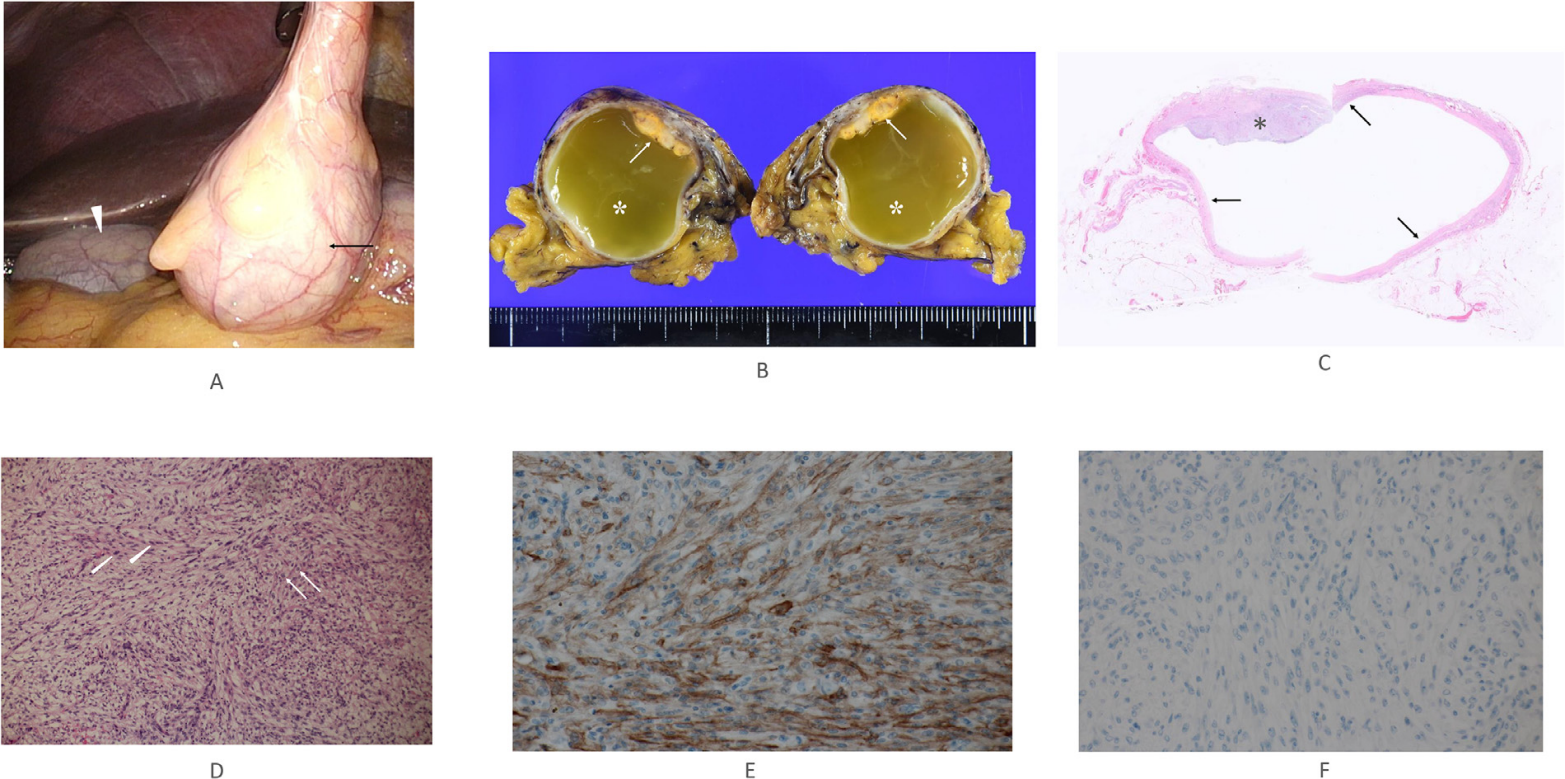
Surgical and pathological findings. (A) Laparoscopy clearly revealed the discontinuation between the gallbladder (arrowhead) and the cystic lesion (arrow). The cystic lesion was located in the round ligament of the liver. (B) Macroscopic view of the bisected mass showed yellowish flat nodule (arrows) and yellow-green liquor in the intracystic tumor (asterisks). (C) Low-magnified view of the intracystic lesion showed a flat type tumor (asterisk) and fibrous cystic wall (arrows). (D) Magnified view of the solid part showed epithelioid cells (arrows) and spindle cells (arrowheads) growing in sheet-like and storiform fashions, respectively. (E) Immunohistochemical staining of the tumor was positive for CD117. (F) Immunohistochemical staining of the tumor was negative for DOG1.

A 44-year-old woman was referred to our hospital for the examination and treatment of a presumed gall bladder tumor. Both ultrasound (US) and computed tomography (CT) showed an intracystic tumor but failed to point out the discontinuity between the cystic lesion and the gallbladder (Fig. 1, Fig. 2). Magnetic resonance imaging (MRI), however, could clearly depict the presumed intracystic tumor and the discontinuity between the gallbladder and the target lesion (Fig. 3). Contents of the gallbladder and the cystic lesion had hypo- and hyper-intense patterns both on T1- and T2-weighted images, respectively. The former, however, had a less hypo-intense pattern on T1-weighted images and a less hyper-intense pattern on T2-weighted images than those of the latter. These findings highly suggested the different liquid contents in them. Positron emission tomography / CT showed no avid radio tracer uptake in the cystic lesion. Endoscopic ultrasonography (EUS) using contrast medium showed a flat mass (Fig. 1B) with homogenous enhancement in the mass component and no invasion beyond the cystic capsule. Under the preoperative diagnosis of early gallbladder cancer despite these image findings, laparoscopic cholecystectomy was attempted to the patient. Laparoscopic observation, however, clearly revealed that the target lesion was not continuous with the gallbladder and was located in the round ligament of the liver (Fig. 4A). Intra-operative findings led us to do cholecystectomy and resection of the adjacent cystic tumor. The intracystic tumor was 3 cm in size and had minute solid component inside the cyst wall (Fig. 4B). Pathological study of the gallbladder showed focal Rokitansky Aschoff Sinus without any atypical cells. The intracystic tumor in the round ligament of the liver pathologically showed a well-demarcated solid tumor in the cyst wall (Fig. 4C). The solid part consisted both of epithelioid cells growing in a sheet like fashion and spindle cells showing a storiform pattern without any necrosis (Fig. 4D). The cystic wall consisted of hypo cellular fibrous tissue and focal complete fibrous tissue (Fig. 4C). Immunohistochemical staining of the tumor was positive for CD117 (Fig .4E) and negative both for desmin and S100, leading to the diagnosis of GIST. MIB-1 labelling index of the GIST was 8%. Postoperative upper and lower gastrointestinal endoscopic examinations showed no GISTs and other solid malignancies in the GI tract. The patient recovered uneventfully and has been well without any recurrences for 3 months.

## Discussion

When examining the gallbladder, US is often used due to its excellent descriptive ability without radiation exposure [8]. However, in this case, wide range multiple reflections of US unfortunately hampered the detailed visualization of the small solid part in the cystic lesion. On the other hand, more detailed evaluation was possible with the EUS due to the absence of multiple reflections of US. However, neither US nor EUS could point out the discontinuity between the gallbladder and the cystic lesion.

CT is useful for evaluating the degree of gallbladder tumor invasion into the liver and regional lymph node swelling [9]. In this case, the gallbladder and the cystic lesion were in close contact over a wide area and was difficult to be negated of their continuity between the 2 even on CT. The difference of CT values (Hounsfield Unit;HU)in the 2 contents was also slight (15HU vs 20HU) and further failed to suggest the discontinuity of the 2 target lesions. Even postoperative retrospective CT re-evaluation could not clarify the discontinuity between the 2 target lesions.

T1- and T2-weighted images of both the gallbladder and the cystic lesion showed hypo- and hyper-intense patterns on T1- and, T2-weighted images, respectively. However, the degree of hypo- and hyper-intense patterns were both somewhat dissimilar, clearly suggesting different properties of the 2 liquid contents. MRI, therefore, is overwhelmingly superior to US and CT in the characteristic evaluation of fluid contents.

Several cystic GISTs have been reported to date [10]. All cystic walls, however, consisted of tumor cells, highly suggesting the pathogenesis of cyst formation to be central necrosis. This hypothesis is supported by the fact that all reported cystic GIST cases were large tumors. However, the cyst wall in this case was mostly composed of fibrous components. In addition, pathological findings clearly negated that the small GIST located in the cyst wall secreted massive liquid. Based on these findings, it is reasonable to interpret that the GIST did not form a cyst, but that the GIST was developed in the cyst wall. Cysts developed in the round ligament of the liver themselves are extremely rare, and GISTs in those cysts are much rarer.

KIT, i.e., CD117 [11], positivity is very important for definitive diagnosis of GISTs. The newly developed DOG1 [12] has better GIST diagnostic ability than that of CD117, and even if CD117 is negative, DOG1 positivity warrants the diagnosis of GIST. Why this case was CD117 positive and DOG1 negative (Fig. 4F) remains uncertain. It has already been proven that the origin of GIST is Cajal cells, being very important cells for peristalsis of the GI tract. Since the diagnostic ability both of CD117 and DOG1 was mainly investigated in GI tract GISTs, it is necessary to verify the usefulness of these 2 immunostainings for GISTs that occur in areas other than GI tract.

## Conclusions

Physicians should note that intracystic GIST can be observed in the round ligament of the liver. MRI is very useful for evaluating the liquid component when diagnosing cystic tumors that have extensive contact with the gallbladder.

## Author contribution

NK designed the concept of this study. SO drafted the manuscript. AF made preoperative image analyses.

## Patient consent

Written informed consent was obtained from the patient for the publication of this case report and any accompanying images.
